# Post-Curing Effects on the Tensile Properties of Hybrid Fiber-Reinforced Polymers: Experimental and Numerical Insights

**DOI:** 10.3390/polym17091261

**Published:** 2025-05-06

**Authors:** Mohammed Zaini, Oumayma Hamlaoui, Jalal Chafiq, Mohamed Ait El Fqih, Mohamed Idiri, Said Aqil, Mohamed Karim Hajji, Alperen Bal, Hakan Tozan, Marta Harnicárová, Jan Valicek

**Affiliations:** 1 Laboratory of Artificial Intelligence & Complex Systems Engineering (AICSE), National Graduate School of Arts and Crafts, Hassan II University, Casablanca 20360, Morocco; mohammed.zaini@capgemini.com (M.Z.); j.chafiq@autohall.ma (J.C.); simohammed.aitelfqih@univh2c.ma (M.A.E.F.); said.aqil@univh2c.ma (S.A.); 2 College of Engineering and Technology, American University of the Middle East, Egaila 54200, Kuwait; mohammad.hajji@aum.edu.kw (M.K.H.); alperen.bal@aum.edu.kw (A.B.); hakan.tozan@aum.edu.kw (H.T.); 3 Laboratory of Engineering and Materials (LIMAT), National Graduate School of Arts and Crafts, Hassan II University, Casablanca 20360, Morocco; mohamed.idiri@univh2c.ma; 4 Department of Electrical Engineering, Automation and Informatics, Faculty of Engineering, Slovak University of Agriculture in Nitra, 949 76 Nitra, Slovakia; marta.harnicarova@uniag.sk (M.H.); jan.valicek@uniag.sk (J.V.); 5 Department of Mechanical Engineering, Faculty of Technology, Institute of Technology and Business in České Budějovice, 370 01 České Budějovice, Czech Republic

**Keywords:** basalt, carbon, composites, epoxy, fiberglass, hybrid, jute, temperature, tensile

## Abstract

This study investigates the effects of post-curing temperatures on the tensile properties of hybrid basalt-jute-glass-carbon fiber-reinforced polymers (FRPs). Composite specimens were post-cured at 60 °C and 100 °C for 60 min, and their tensile behavior was assessed using a servo-hydraulic testing machine. Numerical simulations using the Abaqus software V6.14 were also conducted to compare experimental and computational results. The findings indicate that post-curing heat treatment enhances ductility due to increased polymer cross-linking, but excessive heat treatment at 100 °C negatively impacts elongation at fracture. The results revealed that specimens post-cured at 60 °C exhibited the optimal balance between strength and ductility, with increased elongation and moderate tensile strength. However, at 100 °C, while tensile strength improved in some cases, a significant decrease in elasticity and an increased risk of brittleness were observed, suggesting that extreme heat treatment may degrade polymer integrity. Natural fiber composites, particularly jute-based samples, outperformed synthetic composites in terms of elongation and overall mechanical stability. The numerical simulations provided further insights but showed discrepancies with experimental results, mainly due to fiber property variations and fabric waviness, underscoring the challenges of accurately modeling woven composites. The study highlights the importance of controlled post-curing temperatures in optimizing the mechanical performance of FRP composites, with 60 °C identified as the most effective condition for achieving a favorable balance between tensile strength, flexibility, and material durability. These findings offer valuable insights for material scientists and engineers working on the development of high-performance composite materials for structural and industrial applications.

## 1. Introduction

Post-curing is a critical process in enhancing the mechanical properties of hybrid fiber-reinforced polymers (FRPs). In the context of this study, the term “hybrid” specifically refers to composites that combine two or more distinct types of fibers, such as basalt, jute, glass, and carbon fibers, within a single epoxy matrix to leverage their complementary mechanical properties. It ensures adequate cross-linking within the epoxy matrix, which is essential for improving tensile strength and other mechanical properties. The optimization of post-curing parameters, such as temperature and time, plays a significant role in determining the final properties of the composite materials [1,2,3,4,5]. Several studies have explored the experimental aspects of post-curing on hybrid composites. For instance, research on hybrid ramie and carbon fiber composites demonstrated that temperature had a more significant impact on tensile strength than time. This research evaluated an epoxy system to discover that the combination of 60 °C post-curing temperature for a duration of 12 h presented the best conditions to improve tensile properties based on the specific system [1]. This study employed the Taguchi design of experiments to systematically analyze the effects of post-curing temperature, curing time, and the number of synthetic fiber layers on the overall mechanical performance of the composites. Similarly, increasing post-curing temperatures from 50 °C to 90 °C improved the tensile strength of jute/basalt fiber composites by 45.48%. These findings highlight the importance of carefully selecting post-curing conditions to maximize the mechanical performance of hybrid composites [2].

For instance, Lascano et al. demonstrated that increasing the curing temperature from 70 °C to 90 °C resulted in a substantial increase in tensile strength, highlighting the importance of temperature in achieving optimal mechanical properties [6]. Similarly, Ghani and Mahmud reported that higher post-curing temperatures enhance the shear modulus and elongation of hybrid composites, indicating a positive hybrid effect due to better fiber–matrix interactions [7]. This effect is further supported by the findings of Khalifa et al., who noted that elevated post-curing temperatures improved the mechanical properties of flax fiber-reinforced epoxy biocomposites, suggesting that thermal conditions play a pivotal role in enhancing tensile strength [8].

The hybridization of different fibers also contributes to the mechanical performance of composites. Ismail et al. found that combining kenaf and bamboo fibers improved the tensile properties of epoxy composites, demonstrating the benefits of using hybrid fiber systems [9].

Numerical simulations complement experimental findings by providing insights into the micro-mechanical behavior of composites during post-curing. For example, numerical models have been used to predict the evolution of residual stresses during the curing process, which can affect the fatigue life and tensile properties of FRPs. These models help in understanding the interplay between thermal, chemical, and mechanical factors during curing, allowing for better optimization of the process [10,11].

The mechanical properties of nylon 6/glass fiber-reinforced hybrid composites were studied using different curing methods. Ultraviolet-cured composites exhibited superior mechanical properties compared to thermally cured ones, highlighting the sensitivity of nylon 6 fibers to high-temperature curing and the importance of selecting appropriate curing methods. However, the generated free radicals from UV exposure might damage the composite by reducing its oxidation resistance while simultaneously increasing its brittleness. UV curing results in improved mechanical properties yet long-term material degradation needs thorough examination because of its association with these curing methods [12].

On the other hand, the effect of post-curing heat treatment on the tensile properties of carbon UD/vinyl ester composites using the VARI manufacturing method were investigated by Isna et al. [13]. Their study examined three different post-curing temperatures of 25 °C, 80 °C, and 100 °C and found that increasing the heat treatment temperature significantly improved the ultimate tensile strength (UTS). Compared to composites cured at 25 °C, the UTS increased by 1.17% at 80 °C and 17.46% at 100 °C. However, the standard deviation (SD) or variance data were not explicitly reported in their study, which makes it difficult to quantitatively assess the statistical significance of these improvements. Furthermore, while higher temperatures enhanced tensile strength, they negatively affected stiffness, with the modulus of elasticity decreasing by 7.29% at 80 °C and significantly by 47.42% at 100 °C. These findings suggest a trade-off between strength and stiffness in heat-treated composites and underscore the necessity for detailed statistical data to clearly interpret the practical impact of such small gains.

These findings highlight the trade-off between strength and stiffness in heat-treated composites and emphasize the need for optimal post-curing conditions to balance mechanical properties.

Chemical treatments, such as alkalization and silanization, impact the mechanical properties of natural fiber-reinforced hybrid composites. The treatments can either enhance or diminish tensile, flexural, and impact properties, depending on the fiber type and treatment combination, indicating the complexity of optimizing hybrid composites [14].

The choice of fibers and matrix materials significantly influences the post-curing effects on tensile properties. Studies have shown that hybrid composites, such as those combining natural and synthetic fibers, can achieve superior mechanical properties compared to single-fiber composites. The interaction between different fiber types and the matrix during post-curing can lead to improved tensile strength and other mechanical characteristics [14,15,16].

The mechanical behavior of hemp/sisal fiber-reinforced bioepoxy hybrid composites was studied, showing that different stacking sequences result in variations in tensile strength and other mechanical properties. The intercalated arrangement of fibers provided the highest tensile modulus, demonstrating the importance of fiber arrangement in hybrid composites [17].

The exponentially expanding literature about post-curing effects in fiber-reinforced polymer (FRP) composites primarily studies unidirectional and single-fiber composites instead of blending natural and synthetic fibers together. Research investigating static properties of materials lacks computational models that either validate or predict experimental outcomes in a considerable number of investigations. Multiple studies have missed crucial evaluations of fiber type interconnections and neglect to explain damage processes that occur from extended thermal processing during curing. Research that examines mechanical performance decline tends to neglect how production parameters, including those affecting fiber deformations and interfacial bonds along with fiber arrangement, affect final results. The study deficiencies create obstacles to result generalization and reduce the overall knowledge about hybrid composites during heat processing. A multi-fiber hybrid composite system is subject to systematic experimental and numerical analysis under different post-curing conditions in the present investigation. The study examines how carbon and basalt and glass and jute fiber combinations result in both beneficial and competing effects which enable better understanding of FRP material behavior. Finite element modeling and mechanical response analysis under tensile loads are part of this examination which enables the evaluation of simulation limitations pertaining to modeling actual composite material behavior. This combined experimental-computational method unites experimental study results with computational modeling results to improve understanding about how hybrid composites respond under various post-curing conditions regarding strength, ductility, and durability specifications. Consequently, this research demonstrates both innovation and practical importance. The proposed hybrid composite structures combining natural and synthetic fibers in polymer matrices offer important advantages for versatile engineering applications which require strength improvements with ductility and environmental benefit performance goals. Previous research focused mainly on one-directional composite materials with single fibers but this work establishes new information through multiple fiber types and stacking combinations. The presented research uses experimental and numerical methods to assess the effects of controlled post-curing temperatures as it contributes to a complete comprehension of manufacturing parameter impacts on composite performance. The research is unique because it combines hybridization studies with simulation–experiment correlations which help solve literature deficiencies regarding material optimization and performance prediction.

## 2. Materials and Methods

This section describes the material samples and tests employed. A description of the experiments and simulations is presented.

### 2.1. Specimen Preparation and Characterization

**Figure 1:**
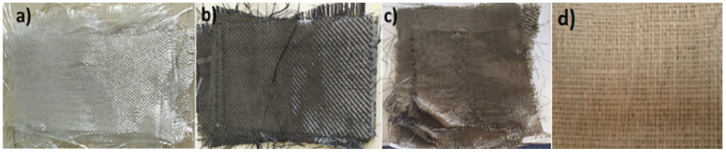
(**a**) Textured glass fiber, (**b**) textured carbon fiber, (**c**) textured basalt fiber, (**d**) textured jute fiber.

In this study, we analyzed the mechanical behavior of five different composite materials. Every one of these materials had a specific set of reinforcing fibers and epoxy parts, leading to composite structures with different characteristics. The materials under study were woven E-glass with a plain weave pattern, the basis weight of which was 2.56 g/cm^2^ and which was supplied by FiberJeely. In addition, UHMW (Ultra-High Molecular Weight) fibers were used as well as basalt fibers in a twill weave pattern which were sourced from Newtex Composites. Carbon reinforcement was performed using twill weave 3k200 fibers from YN Carbon. The epoxy resin of these composites was Atlas Epoxy-based. In order to increase the diversity of the research, the fifth composite material combined jute fibers with a mass per unit area of 367 g/cm^2^, which were supplied by a local manufacturer. The detailed investigation of these features provided us with the possibility to determine how these various types of fibers, weave patterns, and epoxy sources affected the mechanical properties of the composite materials. Figure 1 depicts the diverse fiber weaves utilized in this work, facilitating a visual comparison of each material’s structure and composition.

The composite specimens were fabricated with meticulous attention to detail via the hand lay-up process, a popular approach in the composite manufacturing domain. Particularly, these composites combined basalt-epoxy, glass-epoxy, carbon-epoxy, and basalt-glass-carbon epoxy hybrid types. Each of these composite types was carefully formulated to maintain a consistent weight fraction featuring an equal 50/50 ratio of fibers to epoxy.

To develop a more comprehensive summary of the process, the formation procedure consisted of laminating these composites with nine layers of the polymer and fiber materials. Table 1 provides detailed weight ratios for each composite, presenting a visual illustration of the proportion of fibers and epoxy in each composite type.

Standardized specimens specifically designed for tensile testing were precisely cut from the fabricated laminate plates, following the dimensions prescribed by ASTM D638 [18]. As indicated in Table 2, each sample was manufactured with a width of 13 mm and a length of 165 mm, thus complying with the exact conditions imposed by ASTM D638 [18].

Thorough quality control ensured care was taken in the elements of the manufacturing process to meet industry requirements, and consistency in composition provided that the tensile tests were conducted with accuracy and that the results were reproducible.

To prepare the specimens for testing, they were purposely grouped into three distinct sets, with each set having five specimens. These sets were exposed to different post-curing procedures that were intended to improve the material characteristics. Post-curing steps were implemented with great care to ensure the correctness and dependability of the findings.

The first category of specimens was post-cured at a mild room temperature of 20 °C. This was used as a starting point for evaluating the tensile properties upon elevated thermal treatment.

The approach in the second and third groups was more delimited and detailed. The specimens in the second group were intentionally baked inside an oven at 60 °C for one hour. Concurrently, the samples in the third set were subjected to a similar post-curing procedure except that these were cured at a temperature of 100 °C in the Binder oven for the same one-hour process.

The research adopted temperatures of 20 °C, 60 °C, and 100 °C since these levels corresponded with the practical application requirements along with academic research findings of FRPs. Standard room temperature curing happens at 20 °C, which functions as the control scenario under ambient conditions. The researchers chose 60 °C because the industry widely supports this temperature for epoxy post-curing, which generates effective cross-linking bonds while protecting the polymers from degradation as several previous publications have reported [1,2,3]. The experimental temperature applied ended at 100 °C in order to study how extreme thermal processing affects these natural fibers and prevents degradation of the resin during heating. Experimental practicality and material structural integrity guidance focused the research when working with composite components that combined natural with synthetic fibers. The research temperature limit of 100 °C precludes testing above one hundred degrees Celsius because this may deteriorate the structural composition and thermal resistance of natural fibers including jute and basalt. The chosen set of temperatures enables evaluation of the post-curing conditions, which range from conservative to moderate to elevated, providing an optimal processing strategy for assessing hybrid composite materials.

**Figure 2:**
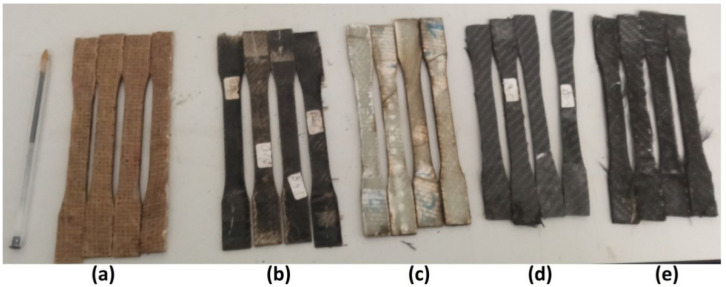
Specimens after cutting: (**a**) JFRP, (**b**) BFRP, (**c**) GFRP, (**d**) CFRP, (**e**) HFRP.

The systematic division and curing enabled conclusions to be drawn about how temperatures and duration affect the tensile properties of the samples. Figure 2 shows the result of this procedure that led to producing five different specimens.

### 2.2. Scanning Electron Microscopy

**Figure 3:**
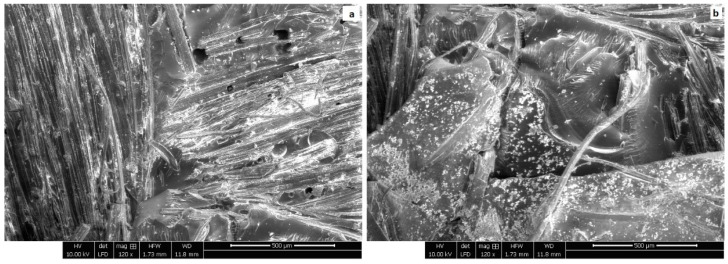
Orientation of the fibers inside bundles in JEC: (**a**) Aligned fiber bundles exhibiting preferential orientation; (**b**) Misaligned and dispersed fiber bundles with evident matrix-rich zones.

**Figure 4:**
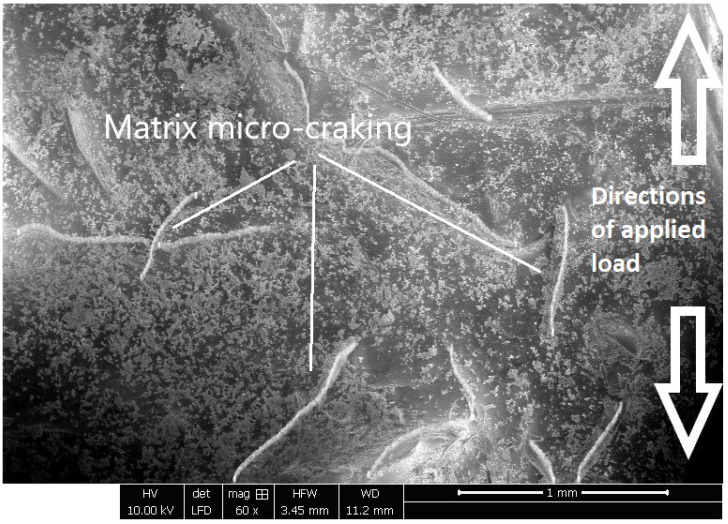
Observations of the microstructure after tensile test for JEC.

The surface adhesion between the jute fibers and the epoxy matrix in the jute epoxy laminate composite was analyzed by scanning electron microscopy (SEM). The outcomes shown in Figure 3 provide information regarding the distribution and direction of the fibers in the bundles of the jute epoxy composite. In addition, the SEM micrograph showed an interesting characteristic of the material. The evidence suggested that the jute fibers in the composite underwent a pulling out phenomenon from the epoxy matrix and showed signs of cracking. This greatly weakened the interfacial bonding of the epoxy matrix and the jute fibers. The decrease in the interfacial adhesion between the jute and epoxy constituents played a significant role in the general mechanical behavior of the composite. The reduced adhesion and interaction resulted in the load-bearing capacity of the jute–epoxy composite dropping, causing structural failures to take place at smaller applied loads, as in the case with the jute composite only illustrated in Figure 4.

### 2.3. Tensile Testing

**Figure 5:**
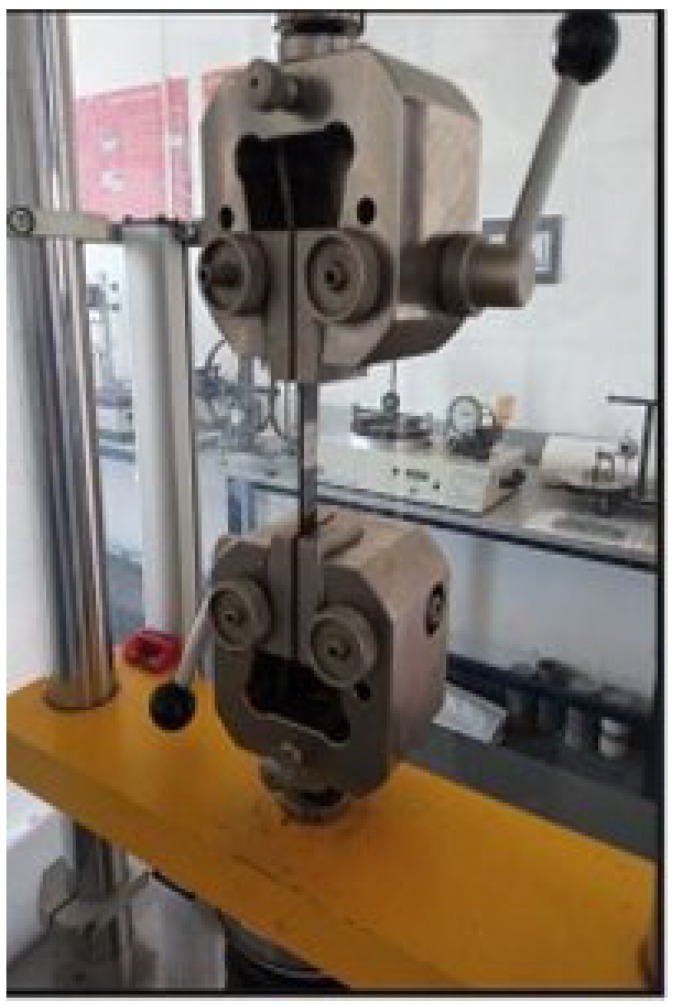
Configuration for conducting tensile tests.

By use of an Instron (Norwood, MA, USA) 4204 universal testing machine, dynamic tensile behavior tests were carried out on the material. The tests followed guidelines adopted by the American Society for Testing Materials (ASTM), which adheres to the ASTM D638 [18] standard testing protocol, as shown in Figure 5. That is why in order to ensure uniformity, a test speed was chosen at a cross-head speed of 60 mm/min for consistency. This testing procedure was applied to five specimens which were tested under different temperature conditions, making the data collection extensive. After the tests were performed, mean values and standard deviations were calculated, creating a strong statistical basis for analysis of the results. This type of calculation aids in understanding the central tendency and variability of the data. A graph of stress and strain in tensile loading, which is a basic representation of the material’s mechanical response under these tests, represents the results of these tests. This graph provides the capability to see and measure important mechanical properties, such as the tensile strength, modulus, and strain at break.

### 2.4. Numerical Simulation

The Finite Element Method (FEM) is widely used and is a reliable method to analyze structures, providing consistent results across various engineering applications [19]. In the context of composite materials, FEM provides an important tool to simulate and forecast their behavior under mechanical loading conditions.

Element analysis software Abaqus, specifically, CAE 6.13-1, was used to perform the numerical evaluations for this research. It offers a flexible framework within which the composite materials can be modeled and analyzed under different mechanical constraints. In this research, for the numerical modeling of tensile specimens, the Abaqus CAE 2017 software version was used.

Using the capabilities of Abaqus and the finite element method, numerical simulations were performed that augmented the experimental tests, providing a complete assessment of the behavior and performance of the composites subjected to tensile loading. The combination of experimental and numerical methods can improve the general validity and depth of the research results, making them more useful for engineering and materials science purposes.

## 3. Results and Discussion

This section presents the experimental and numerical findings on the tensile properties of various fiber-reinforced polymer (FRP) composites. The impact of the post-curing temperature on mechanical behavior is analyzed in detail, providing insight into material performance variations due to thermal treatment. The results are systematically compared to theoretical predictions and numerical simulations to validate the observed trends.

### 3.1. Results

#### 3.1.1. Tensile Properties

**Figure 6:**
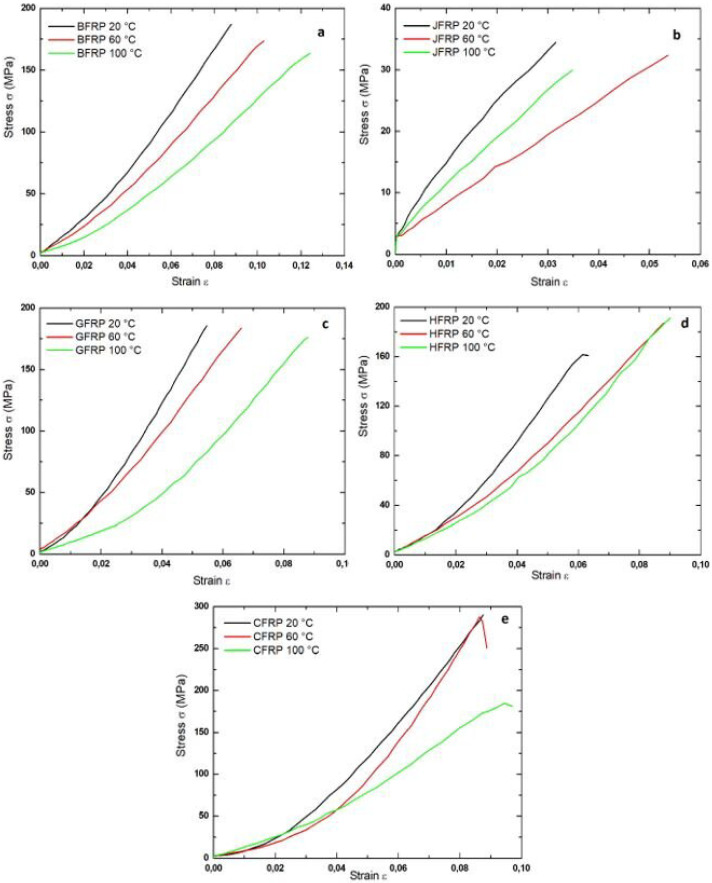
Stress–strain curve of different composites treated at different temperatures (**a**) BFRP, (**b**) JFRP, (**c**) GFRP, (**d**) HFRP, (**e**) CFRP (20 °C, 60 °C, and 100 °C).

Four different composite materials: basalt/epoxy, jute/epoxy, glass/epoxy, and carbon/epoxy were tested and their physical and mechanical characteristics are presented in Table 3. These properties were used for computational modeling. Our analysis measures Young’s modulus (*E*) values in multiple directions, plus the shear modulus (*G*) and Poisson’s ratio (*v*). Experimental tests and rule of mixtures analytics produced these material values. The basalt/epoxy laminate displays greater stiffness than jute because it has a higher modulus of elasticity at 206 GPa against 104 GPa for jute. The carbon/epoxy laminates maintain superior resistance to change of shape from shear forces with a top shear modulus value of 157 GPa. The jute laminates exhibit the greatest lateral expansion during axial loading with a Poisson’s ratio of 0.08. The study illustrates how materials behave differently and sets up the base requirements for both computer simulations and physical experiments with these composites. In Figure 6, the tensile properties of the BFRP, CFRP, GFRP, and JFRP materials are given at three different post-cure temperatures (20 °C, 60 °C, 100 °C). The ultimate strength decreased from 187 MPa to 163 MPa, and the elongation at break increased from 8.79% to 12.4%. The ultimate strength for JFRP did not change perceptibly. Nevertheless, maximum elongation at break is achieved at 60 °C. For the JFRP, the result for temperatures of 20 °C and 60 °C is the same, whereas at 100 °C, the elongation at break increased from 5.47% to 8.78%. The performance of CFRP does not change at 60 °C. The elongation at break changed from 8.75% to 9.7%. The ultimate strength was reduced to only 181 MPa from its previous level of 290 MPa. The hybridization of the fiber (basalt, carbon, glass) generally causes the ultimate strength to increase from 190 MPa to 161 MPa. An improved result was observed for elongation that grew from 6.33% to 9%, as shown in Table 4. The ultimate elongation at break and the strength of the material reduced with increase in temperature of heat treatment as the results demonstrate. Table 4 contains mechanical data regarding fiber-reinforced polymer (FRP) composites which received post-curing treatment at three different temperatures (20 °C, 60 °C, and 100 °C). Tensile strength tests on carbon fiber-reinforced polymers (CFRPs) yielded 290 MPa measured at 20 °C room temperature but jute fiber-reinforced polymers (JFRPs) achieved minimum tensile strength, ranging between 30 and 34 MPa under all curing conditions. The BFRP and GFRP tensile strength diminished slightly when post-curing temperatures increased yet their elongation at break values rose, leading to better ductility. The higher cross-link density developed within epoxy resin at elevated temperatures promotes better energy absorption during failure. The stiffness of BFRP showed a substantial decline at 100 °C because its Young’s modulus decreased from 206 GPa to 13 GPa, which could indicate thermal degradation or that interfacial damage may occur when curing temperatures become excessive. The mechanical properties of the HFRP composites remained balanced through the entire testing temperatures while achieving strong tensile strength and elongation levels at 60 °C. The results demonstrate the profound impact of the post-curing temperature on FRP composite mechanics since it determines how matrix and fiber interaction result in optimal strength and flexibility characteristics. The tensile strength measurements from jute fiber-reinforced polymer (JFRP) composite tests reached 30–34 MPa. These results fell below standard jute-epoxy composite strength ranges established in the literature, which span from 50 to 100 MPa. Various factors seem to have caused this deviation. The source jute fibers remained untreated because the supplier did not provide chemical treatment like alkalization or silanization, although these processes enhance bonding between fibers and the matrix. The interface between fiber and matrix showed inadequate bonding behavior because it caused early detachment of fibers; this observation matches the SEM images which reveal separation of fibers and matrix cracks. Manual hand lay-up manufacturing as a prototyping technique leads to inconsistent fiber distribution and void contents because operators apply minimal compaction pressure during the manual processing steps. Natural jute fiber properties that vary in terms of diameter size and alignment pattern along with moisture absorption characteristics directly impact the mechanical strength. The uncoated composite structure with nine stacked layers lacked through-thickness reinforcement and interlayer stitching because these features would restrict tensile load resistance. Multiple combined influences seem to have caused the reported decrease in tensile strength for jute composites in laboratory settings that utilize ideal or chemically processed jute. The results suggest that subjecting FRP composites to heat curing post-treatment has a positive effect on the impact toughness and elongation. Elevated tempering at 60 °C effectively boosts composite ductility as optimal cross-linking occurs but it remains crucial to note that excessive cross-linking which occurs at temperatures reaching 100 °C restricts free polymer chain movement. These limitations lead to reduced elongation at fracture while causing brittleness because post-treatment reduces the flexibility of molecular chains. The post-curing temperature needs precise control because it determines the ideal strength–ductility relationship in the material. This improvement is due to the reinforced composite which is developed by the better cross-linking in the epoxy polymer matrix during the post-curing stage. The exponentially expanding literature about post-curing The study conducted confirms that post-curing treatment results in substantial mechanical changes which weaken both the tensile strength and stiffness in fiber-reinforced polymers (FRPs). The primary cause of such degradations originates from micro-defects forming at the fiber–matrix interfaces, which result from both resin and reinforcement fiber thermal expansion coefficient mismatches as well as curing cycle thermal fluctuations. A weakened bond between the interfaces leads to reduced stiffness because the load transfer effectiveness suffers at these areas. During loading, the interface disruption causes stress redistribution that leads to increased elongation at fracture, thus helping to compensate for the tensile strength decrease and improving ductility. However, it is important to note that such disruption of the interface, while reducing tensile strength, can simultaneously contribute to increased elongation at fracture. This phenomenon is supported by micromechanical theories, which suggest that weak fiber–matrix adhesion can enable localized interfacial debonding and fiber pull-out during loading. These mechanisms dissipate energy and delay the onset of catastrophic failure, thereby allowing the material to undergo more deformation before rupture. In particular, the increased ductility observed in post-cured composites may result from these interfacial imperfections acting as crack deflection paths, which slow crack propagation and enhance strain accommodation. This behavior is consistent with previously reported findings on natural and hybrid composites, where moderate interface weakening improved toughness and ductility, despite a reduction in peak load capacity. The interruption redirects the energy from any impact or load into the creation of many tiny cracks, which are an energy-dissipating mechanism. The process has the benefit of preventing macroscopic cracks developing because the cracks will grow through the body of the material and catastrophic failure will take place. Basically, the post-curing process results in a trade-off between tensile strength and elongation at break in FRP materials. When the interface is interrupted, the tensile strength decreases, but the improved capability for energy dissipation and crack deviation leads to greater elongation when the fracture occurs.

#### 3.1.2. Numerical Analysis

Nowadays, it is very challenging to find a model of law behavior for composite materials. Few studies have been conducted on this subject. Hamlaoui et al. investigated the influence of glass fiber content on the mechanical and physical properties of polybutylene terephthalate (PBT) composites, utilizing experimental analysis to predict mechanical responses under tensile and flexural loads to correlate the fiber content to the mechanical properties [20]. Their study incorporated micromechanical modeling techniques to simulate fiber–matrix interactions and validate experimental findings. Similarly, Bounjoum et al. explored damage patterns in carbon fiber-reinforced polymers (CFRP) through both simulation and experimentation, employing progressive damage models based on continuum damage mechanics (CDM) [21]. Their approach integrated fracture mechanics principles to analyze crack propagation and failure initiation under cyclic and impact loading, further demonstrating the complexities of accurately predicting composite material behavior.

In their study, Valíček et al. emphasized the importance of precise modeling in understanding the mechanical behavior of materials subjected to external stresses, particularly those processed by abrasive waterjet (AWJ) technology. By developing a method that quantifies stress-deformation relationships based on surface topography analysis, they provided a non-destructive approach to determining key mechanical properties, such as Young’s modulus, yield strength, and ultimate strength. This modeling framework enhances the predictive capabilities for material performance, allowing improved optimization of machining processes and structural integrity assessments [22].

In this study, we will use the Johnson–Cook model for predicting the mechanical behavior of hybrid fiber-reinforced composites under different post-curing conditions, considering strain rate sensitivity, work hardening, and thermal softening effects to accurately simulate their tensile response.

Before explaining the approach followed, a reminder of the law is necessary. The Johnson–Cook law is a law of behavior of metallic materials in the nonlinear domain given by (1).

(1) $$\sigma =\left(A+B\epsilon^{n}\right)\left(1+C\ln\left(\frac{\dot{\epsilon }}{{\dot{\epsilon }}_{0}}\right)\right){\left(1-\frac{T-T_{a}}{T_{F}-T_{a}}\right)}^{m}$$

where ϵ is the deformation, $T_{a}$: ambient temperature, $\dot{\epsilon }$: strain rate, $T_{f}$: melting temperature, ${\dot{\epsilon }}_{0}$: reference strain rate, and *A*, *B*, *C*, *n*, *m*: the model parameters.

The first part of the equation addresses the deformation due to work hardening. The second translates the effect of the strain rate. The third part considers the effect of the thermal properties on mechanical behavior. Taking the Johnson–Cook law out of context and applying it to the studied composite material is one of the puroses of this research. A rewriting of the law (1) is necessary to consider the previous hypotheses, giving (2) for the elastic zone and (3) for the plastic zone.

(2) $$\sigma =E\epsilon_{el}$$

(3) $$\sigma =\left(A+B\epsilon_{pl}^{n}\right)\left(1+C\ln\left(\frac{\dot{\epsilon }}{{\dot{\epsilon }}_{0}}\right)\right)$$

*E*, *A*, *B*, *C*, *n* are the parameters to be identified experimentally. The parameters were identified using the direct method. This consists of identifying the parameters of the model term-by-term from the experimental curves. Calculation of the Young’s modulus *E* was conducted as a first step in the identification process. This involves drawing a linear trend curve in the elastic zone and deducing the slope of the curve. Another calculation was necessary in this process, which is calculation of the *A* parameter. Theoretically, the parameter *A* represents the elastic limit of the material, which has been directly identified from the curves.

According to the literature, the elastic limit of short-fiber composite materials is generally between 30% and 33% of the ultimate tensile strength Rm (UTS), which should perfectly align with our findings. According to the ISO 527-1 standard [23] specifying the general principles for the determination of the tensile properties of plastics and plastic composites, the standard deviations and confidence intervals are given at 95%, thus the tolerance interval is equal to ±5% of the arithmetic mean value. The idea is then to calculate the relative error between the experimental curve and the elastic curve. The elastic limit (4) is the stress for which the relative error exceeds 5%.

(4) $$\epsilon \%=\frac{\epsilon_{exp}-\epsilon_{elas}}{\epsilon_{elas}}\cdot 100$$

For the *n* parameter, quasi-static tests were carried out and the constitutive law is reduced in the plastic zone to the below formula (5).

(5) $$\sigma =A+B\epsilon_{pl}^{n}$$

Since we have the below:

(6) $$\left\{\begin{array}{c} \sigma_{i}={\left(A+B\epsilon_{pl}^{i}\right)}^{n}\\ \sigma_{j}={\left(A+B\epsilon_{pl}^{j}\right)}^{n} \end{array}\right.$$

The indices *i* and *j* are used to indicate two different data points along the experimental curve after yielding, and are selected to facilitate calculation of the strain hardening exponent *n* using logarithmic relationships in subsequent equations.

Then,

(7) $$\frac{\sigma_{i}-A}{\sigma_{j}-A}={\left(\frac{\epsilon_{pl}^{i}}{\epsilon_{pl}^{j}}\right)}^{n}$$

with

(8) $$n=\frac{\ln\left(\frac{\sigma_{i}-A}{\sigma_{j}-A}\right)}{\ln\left(\frac{\epsilon_{pl}^{i}}{\epsilon_{pl}^{j}}\right)}$$

(9) $$\epsilon_{pl}^{i}=\epsilon_{i}-\frac{\sigma_{i}}{E}$$

The calculation of the *B* parameter is carried using the plastic zone of the curve based on (6), which gives:

(10) $$B=\frac{\sigma_{i}-A}{\epsilon_{pl}^{n}}$$

The calculation of the *C* parameter is conducted by analysis of the tests results at different strain rates. The strain control in the plastic zone makes it possible to write the following (11):

(11) $$\left\{\begin{array}{c} \sigma_{i}=\left(A+B\epsilon_{pl}^{n}\right)\left(1+C\ln\left(\frac{{\dot{\epsilon }}_{i}}{{\dot{\epsilon }}_{0}}\right)\right)\\ \sigma_{j}=\left(A+B\epsilon_{pl}^{n}\right)\left(1+C\ln\left(\frac{{\dot{\epsilon }}_{j}}{{\dot{\epsilon }}_{0}}\right)\right) \end{array}\right.$$

which gives (12):

(12) $$C=\frac{\sigma_{j}-\sigma_{i}}{\sigma_{i}\cdot \ln\left(\frac{{\dot{\epsilon }}_{j}}{{\dot{\epsilon }}_{0}}\right)-\sigma_{j}\cdot \ln\left(\frac{{\dot{\epsilon }}_{i}}{{\dot{\epsilon }}_{0}}\right)}$$

**Figure 7:**
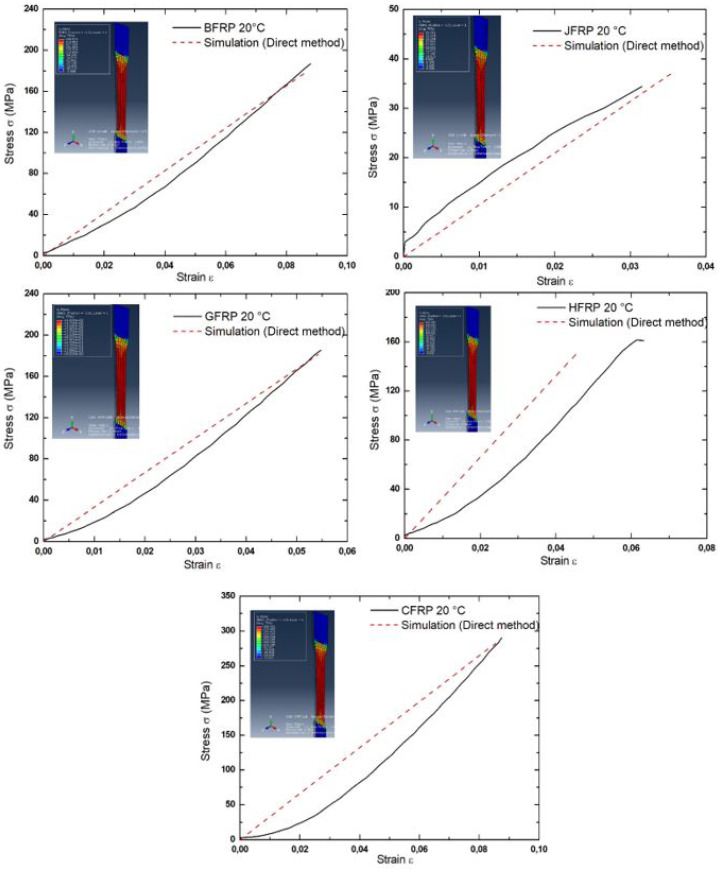
Comparison of simulation and experimental curves.

In Figure 7, a detailed comparison of the simulation curve and the experimental curve is given. This comparative analysis helps us to determine concordances and differences between the two datasets. It should be underlined that in this case, an error of more than 10% was recorded. The main reason why there is such a significant difference between the experimental and numerical results is the natural wavy pattern of the reinforcement materials. The waviness or curvature that occurs in the fibers within the weave structure is a difficult factor to predict from the point of view of the behavior of the material. These fibers load-straightening provide a source of additional displacement which is not always straightforward to accurately model. The observation of this error originating from waviness highlights the difficulty of simulating real-world material behavior, particularly in woven composites. Even though numerical simulations are useful for approximation of behavior, they do not enable detailed analysis of the behavior of complex weave patterns and the interactions occurring between the fibers. This implies the need for continued improvement and verification of numerical models to capture these complexities, leading to better predictions and understanding of composite material behavior. The fracture stress of BFRP is 179.614 MPa, at which point it shows elongation at break of 8.68%. JFRP fails at 37.161 MPa and has elongation at break at 3.55%. GFRP breaks at 181.405 MPa and has elongation at break at 5.42%. Furthermore, CFRP crashes at 282.388 MPa and exhibits elongation at break at 8.54%. Lastly, HFRP fails at 151.399 MPa and has elongation at break at 4.58%. In addition, in the woven reinforcements structures, the fibers of the weave are under tensile and shear stresses when the composite material is subjected to tensile loading. This dual stress effect is responsible for the relatively poor tensile strength exhibited by these materials. Typically, in numerical simulations, perfect bonding between plies is assumed, making the model easier to analyze. But in real-life cases, there is usually a phenomenon of delamination between plies. Delamination occurs when the layers of composite material start to peel away from each other when loads are applied, forming gaps or voids between the plies. This effect may significantly alter the behavior of the composite material. The differences between the experimental and the numerical simulation results beyond 10% error mostly stem from the woven fiber reinforcement materials showing waviness and geometric irregularities while being used to create the composite specimens. The stress concentrations from both fiber misalignment and crimp, together with interlacing patterns, require detailed modeling since it is difficult to achieve high precision modeling in numerical simulation tools. Further studies must conduct quantitative assessments using microscopy methods with automated image analysis to support the identified assumption about fiber waviness.

The numerical model in this study uses homogenized material properties while also assuming perfect ply interfacial bonding, thus disregarding features like matrix cracking and interlaminar delamination and fiber-matrix debonding. The simplifications made to the modeling structure though useful for lowering computational difficulty result in limited mechanical behavior simulation of hybrid fiber-reinforced composites during tensile loading at the mesoscale. The experimental observations deviate from the simulation outputs mainly because simulated progressive damage modeling and in-plane shear deformation mechanisms are absent. Improved future simulations require the incorporation of constitutive parameters derived from experiments, geometric imperfect features, along with damage evolution equations at the microscopic level into the modeling system. It is important to appreciate the dissimilarities between simulation assumptions and the actual structural response. It underscores the necessity of incorporating delamination effects into modeling techniques to bring simulations closer to reality. By resolving this complexity, stronger and more realistic numerical models can be designed. Such models will better reflect woven composite material behavior and help in determining the best application of such products in many industries.

### 3.2. Discussion

In this study, the effect of the post-cure temperature on the tensile properties of various fiber-reinforced polymers (FRPs) and modern composites was investigated, and it was found that at high temperatures, a significant increase in ductility was obtained. This is consistent with the findings of Ouchte et al. [24], who showed that the mechanical properties of jute/epoxy composites improved following 60 °C treatment in comparison to both room temperature and 100 °C treatments. Under the same conditions, Chafiq et al. [25] also noted that hybrid composites with basalt, glass, and carbon fibers exhibited increased ductility in relation to the same parameters. However, the findings of our study slightly deviate with respect to tensile strength; post-curing at 120 °C significantly improved the tensile strength of jute–banana–glass fiber composites [26], while Ramesh et al. [26] showed in their study that the tensile strength of composites decreased with increasing post-curing temperature. This variation is also supported by the study of a number of fibers and composite structures. In addition, Chavan et al. [27] noted that post-curing could enhance synthetic fibers capacity to naturally bind, which is congruent with our finding of an increase in the reactivity of the epoxy matrix. Uzay et al. [3] claimed that post-curing usually enhances mechanical properties, but the influence depends on the composition of the composite material and can be quite different. This was also pointed out by Khalid et al. [28], who found that the predictability of numerical modeling in the tensile response evaluation of these types of complex materials is similar to our FEM supplement to physical testing. On the other hand, the overall literature consensus, which includes the findings of Ouchte et al. [24] and Ramesh et al. [26], is in line with our results that post-curing processes may decrease the tensile strength but enhance other mechanical properties such as elongation and toughness. The study findings agree with existing research outcomes and present additional information about these results. Post-curing at moderate temperatures in the research of Ouchte et al. [24] and Chafiq et al. [25] resulted in improved ductility and maintained tensile strength within natural and hybrid fiber systems. The optimal post-curing temperature at 60 °C provides suitable results for diverse composites but jute-based materials experience reduced strength and increased flexibility at 100 °C heat treatment. The research findings of Ramesh et al. [26] support their statement that higher temperatures for post-curing will degrade the matrix material leading to decreased mechanical performance. This research stands distinct by modeling detailed finite element simulations of hybrid composites in addition to experimental validations through an extensive composite spectrum, thus providing extensive insight into the thermal effects on multimaterial structures. These comparisons highlight the distinctive aspects of our study concerning combining methodologies and wide-ranging material analysis which leads to enhanced thermal–mechanical knowledge of hybrid FRPs.

## 4. Conclusions

The post-curing temperature effects on the tensile properties of hybrid fiber-reinforced polymers (FRPs) that contain natural and synthetic fibers were evaluated. Multiple tests through experimentation and numerical simulation examined different composite types, including BFRP, JFRP, GFRP, CFRP, and HFRP, which were subject to post-curing temperatures at 20 °C (ambient), 60 °C, and 100 °C. The mechanical properties of composite materials were optimally balanced after post-curing at 60 °C because this temperature boosted ductility without substantially decreasing tensile strength. The mechanical characteristics of natural fiber composites suffered damage after excessive heating at 100 °C since the elasticity and stiffness levels decreased significantly due to suboptimal curing practices.

The research undertaken established that the polymer matrix reaches a critical thermal point where structural breakdown occurs, thus lowering its mechanical stability. Post-curing temperatures exceeding 100 °C caused jute-based composites to stretch more but lose their strength and stiffness simultaneously. HFRP hybrid composites obtained advantages from multiple fiber characteristics but their performance was not optimal because thermal stress-induced interfacial failures and fiber-matrix debonding occurred.

The finite element simulation approaches proved useful for prediction but failed to accurately duplicate actual mechanical reactions because they depend on assumptions regarding bonded perfection and material homogeneity. Accurate modeling of hybrid woven composites needs sophisticated approaches that can handle fiber waviness together with damage initiation and progressive failure mechanisms due to experimental and numerical simulation results having variations beyond 10%.

The research adds value to knowledge of composite materials by providing extensive post-curing hybrid FRP evaluations while evaluating model limitations in this field. The experimental data confirm the essential requirement to select appropriate post-curing operational parameters that match the particular fiber compositions and required material properties. This research work suggests future possibilities for quantitative microstructural examinations as well as advanced damage modeling and multi-scale simulation to improve predictive tools in composite engineering.

## Figures and Tables

**Table 1:** Sequencing of samples’ layers and their respective mass proportions.

Samples Stacking Sequence	Coding	Weight of Fibre (Wf) (g)	Weight of Polymer (Wp) (g)	Weight of Hardener (g)
Basalt/Basalt/Basalt				
Basalt/Basalt/Basalt				
Basalt/Basalt/Basalt	BFRP	85	56.66	28.33
Jute/Jute/Jute				
Jute/Jute/Jute				
Jute/Jute/Jute	JFRP	79	52.30	26.60
Glass/Glass/Glass				
Glass/Glass/Glass				
Glass/Glass/Glass	GFRP	134	89.33	44.66
Carbon/Carbon/Carbon				
Carbon/Carbon/Carbon				
Carbon/Carbon/Carbon	CFRP	86	57.33	28.66
Carbon/Glass/Basalt				
Carbon/Glass/Basalt				
Carbon/Glass/Basalt	HFRP	99	66.00	33.00

**Table 2:** Samples’ dimensions (ASTM D638 [18]).

Samples Stacking Sequence	Coding	Average Thickness (mm)	Gauge Length (mm)	Width (mm)
Basalt/Basalt/Basalt				
Basalt/Basalt/Basalt				
Basalt/Basalt	BFRP	2.50	50	13
Jute/Jute/Jute				
Jute/Jute/Jute				
Jute/Jute/Jute	JFRP	3.10	50	13
Glass/Glass/Glass				
Glass/Glass/Glass				
Glass/Glass/Glass	GFRP	2.88	50	13
Carbon/Carbon/Carbon				
Carbon/Carbon/Carbon				
Carbon/Carbon/Carbon	CFRP	2.80	50	13
Carbon/Glass/Basalt				
Carbon/Glass/Basalt				
Carbon/Glass/Basalt	HFRP	2.90	50	13

**Table 3:** Characteristics for computational modeling.

Physical Property	Basalt/Epoxy Lamina	Jute/Epoxy Lamina	Glass/Epoxy Lamina	Carbon/Epoxy Lamina	Source
$E_{1}=E_{2}$ (MPa)	2060	1040	3330	3300	Experimentally
$E_{3}$ (MPa)	2005	990	3002	3200	Analytically (rule of mixture)
$G_{12}$ (MPa)	1070	560	1520	1574	Analytically
$G_{23}=G_{13}$ (MPa)	1020	510	1508	1536	Analytically (rule of mixture)
$v_{12}$	0.065	0.08	0.07	0.045	Experimentally
$v_{23}=v_{13}$	0.32	0.30	0.32	0.30	Analytically (rule of mixture)

**Table 4:** Mechanical properties of the studied composites.

Samples Coding	Young’s Modulus *E* (GPa)	Tensile Strength $R_{m}$ (MPa)	Elongation at Break *A* (%)
BFRP 20 °C	206	187	8.79
BFRP 60 °C	165	174	10.30
BFRP 100 °C	13	163	12.40
JFRP 20 °C	104	34	3.16
JFRP 60 °C	56	32	5.36
JFRP 100 °C	8	30	3.48
GFRP 20 °C	333	185	5.47
GFRP 60 °C	269	183	6.60
GFRP 100 °C	194	176	8.78
CFRP 20 °C	33	290	8.75
CFRP 60 °C	314	250	8.87
CFRP 100 °C	194	181	9.70
HFRP 20 °C	25	161	9.00
HFRP 60 °C	20.38	187.18	8.79
HFRP 100 °C	20.72	190.95	6.33

## Data Availability

The original contributions presented in this study are included in the article. Further inquiries can be directed to the corresponding author.
